# Tuberculous Infection of a Posterior Mediastinal Neuroenteric Cyst Presenting as Secondary Autoimmune Hemolytic Anemia: A Case Report

**DOI:** 10.7759/cureus.113787

**Published:** 2026-08-01

**Authors:** Asvar Samaa, Hibba Kokab, Muhammad Bilal Mirza, Syed Muhammad Javed Iqbal, Syeda Maria Javed

**Affiliations:** 1 Pediatric Medicine, The Children's Hospital & University of Child Health Sciences, Lahore, Lahore, PAK; 2 Pediatric Gastroenterology, The Children's Hospital & University of Child Health Sciences, Lahore, Lahore, PAK; 3 Pediatric Surgery, Children Hospital, Faisalabad, Faisalabad, PAK; 4 Pediatric Surgery, The Children’s Hospital and University of Child Health Sciences, Lahore, Lahore, PAK; 5 Pediatric Medicine, The Children’s Hospital and University of Child Health Sciences, Lahore, Lahore, PAK

**Keywords:** autoimmune hemolytic anemia, neuroenteic cyst, pediatric tuberculosis, posterior mediastinum, tuberculosis

## Abstract

Childhood tuberculosis (TB) can present with atypical and rare manifestations, posing significant diagnostic challenges. While mediastinal masses in children are frequently malignant, congenital cysts can become secondarily infected. Furthermore, TB is a rare but recognized cause of secondary autoimmune hemolytic anemia (AIHA).

We present the case of a four-year-old partially vaccinated boy from Lahore who presented with a four-month history of low-grade fever, progressive pallor, and jaundice. Clinical examination revealed severe pallor, icterus, and hepatosplenomegaly. Laboratory investigations confirmed a Coombs-positive hemolytic anemia. Imaging revealed a large cystic lesion in the posterior mediastinum with vertebral anomalies, initially raising suspicion for a malignancy. The patient underwent surgical excision of the cyst, which yielded purulent fluid. Histopathology confirmed a neuroenteric cyst with chronic granulomatous inflammation and necrosis. The patient was initiated on anti-tuberculous therapy (ATT), resulting in rapid clinical and hematological improvement.

This case highlights an extremely rare triad of a congenital neuroenteric cyst, secondary TB infection, and AIHA. In TB-endemic regions, a high index of suspicion must be maintained for atypical presentations of TB, including superinfection of congenital anomalies and unusual hematological complications. Multidisciplinary coordination is vital for accurate diagnosis and management.

## Introduction

Tuberculosis (TB) remains a critical public health crisis in endemic regions. According to the WHO Global TB Report 2023, Pakistan ranks fifth among high-burden TB countries, with childhood TB constituting approximately 14% of all notified cases [1]. While pulmonary TB is the most common presentation, extrapulmonary and atypical manifestations frequently pose diagnostic dilemmas.

Mediastinal masses in children are predominantly malignant (70-75%), with congenital cystic lesions accounting for only 7% of cases [2]. Neuroenteric cysts (NECs) are exceptionally rare congenital anomalies resulting from incomplete separation of the notochord and foregut, constituting less than 1% of mediastinal masses [3]. While usually asymptomatic or presenting with mechanical compression, these cysts can become secondarily infected.

Furthermore, hematological abnormalities associated with TB are common, but Coombs-positive autoimmune hemolytic anemia (AIHA) secondary to TB remains one of the rarest manifestations, with only a few case reports in the literature [4]. We present a unique case of a posterior mediastinal NEC infected with *Mycobacterium tuberculosis*, presenting with secondary AIHA, emphasizing the concept of "common presenting as uncommon."

## Case presentation

A four-year-old boy, a resident of Lahore, presented to the emergency department with a four-month history of low-grade intermittent fever (documented up to 100°F), progressive pallor, and yellowish discoloration of the skin and eyes. The pallor was initially mild but became pronounced over time and was associated with easy fatiguability and decreased playfulness. The mother also reported weight loss and decreased appetite. There was no history of chronic cough, dyspnea, abdominal pain, bleeding from any site, joint pains, or altered sensorium.

His past history was significant for pyomeningitis at the age of two years, which was treated for two weeks. He was born to consanguineous parents and was the only child, with no history of sibling deaths or miscarriages. The child's vaccination record was unavailable. According to parental recall, he had not completed the primary immunization series. The BCG scar was absent, but whether this reflected non-vaccination or failure of scar formation could not be determined retrospectively. There was no known history of TB contact.

On examination, the child was conscious and cooperative but thin and lean. Vitals revealed a pulse rate of 120/min, respiratory rate of 30/min, blood pressure of 90/60 mmHg, temperature of 100°F, and oxygen saturation of 98% on room air. Anthropometry showed a weight of 10 kg, height of 95 cm, and mid-upper arm circumference (MUAC) of 11 cm, indicating severe acute malnutrition. Systemic examination revealed severe pallor and icterus. There was no lymphadenopathy, petechiae, or stigmata of chronic liver disease. Abdominal examination revealed soft, non-tender hepatomegaly (liver palpable 3 cm below the right costal margin) and splenomegaly (spleen palpable 6 cm below the left costal margin and firm in consistency). Cardiorespiratory and central nervous system examinations were unremarkable.

Initial laboratory investigations showed a dimorphic blood picture on peripheral smear (mixed microcytic hypochromic and macrocytic red blood cells), with adequate white blood cells and platelets and no blast cells. The reticulocyte count was 4%, and the erythrocyte sedimentation rate (ESR) was elevated at 65 mm/hour. Serum lactate dehydrogenase (LDH) and total and indirect bilirubin levels were consistent with hemolysis. The direct Coombs test was positive. Further workup to ascertain the cause of the mediastinal mass and hematological anomaly revealed an alpha-fetoprotein level of 2.1 mcg/L (normal <40), beta-hCG level of <2.3 IU/L (normal <50), normal immunoglobulin levels, negative HIV screening, and negative blood cultures. The stool GeneXpert test was negative. Abdominal ultrasonography confirmed hepatosplenomegaly with normal renal parenchyma.

**Figure 1:**
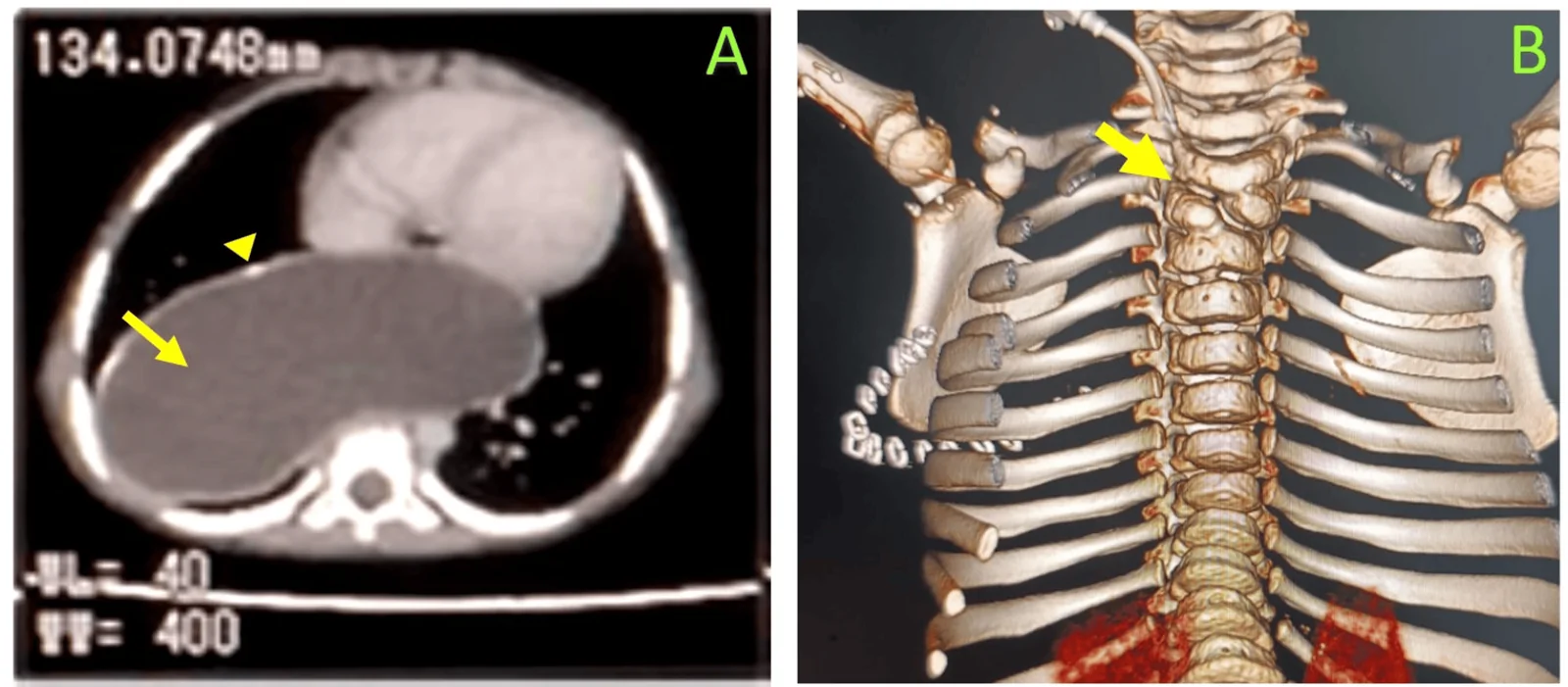
Contrast-enhanced CT and three-dimensional reconstruction. (A) Axial contrast-enhanced CT demonstrating a large posterior mediastinal cystic lesion (arrow) with peripheral rim calcification (arrowhead), extending into the prevertebral region and displacing the soft tissue structures (B) Three-dimensional CT reconstruction demonstrating congenital vertebral anomalies involving the T1 and T2 vertebrae (arrow).

A chest X-ray demonstrated mediastinal widening with a right-sided irregular mediastinal mass. A contrast-enhanced CT scan of the chest and abdomen revealed a 12 × 10 × 6 cm cystic lesion with rim calcification in the posterior mediastinum, occupying the right hemithorax and extending into the prevertebral region and retroperitoneum, displacing the esophagus anteriorly (Figure 1A). Congenital vertebral anomalies at T1 and T2 were also noted (Figure 1B).

**Figure 2:**
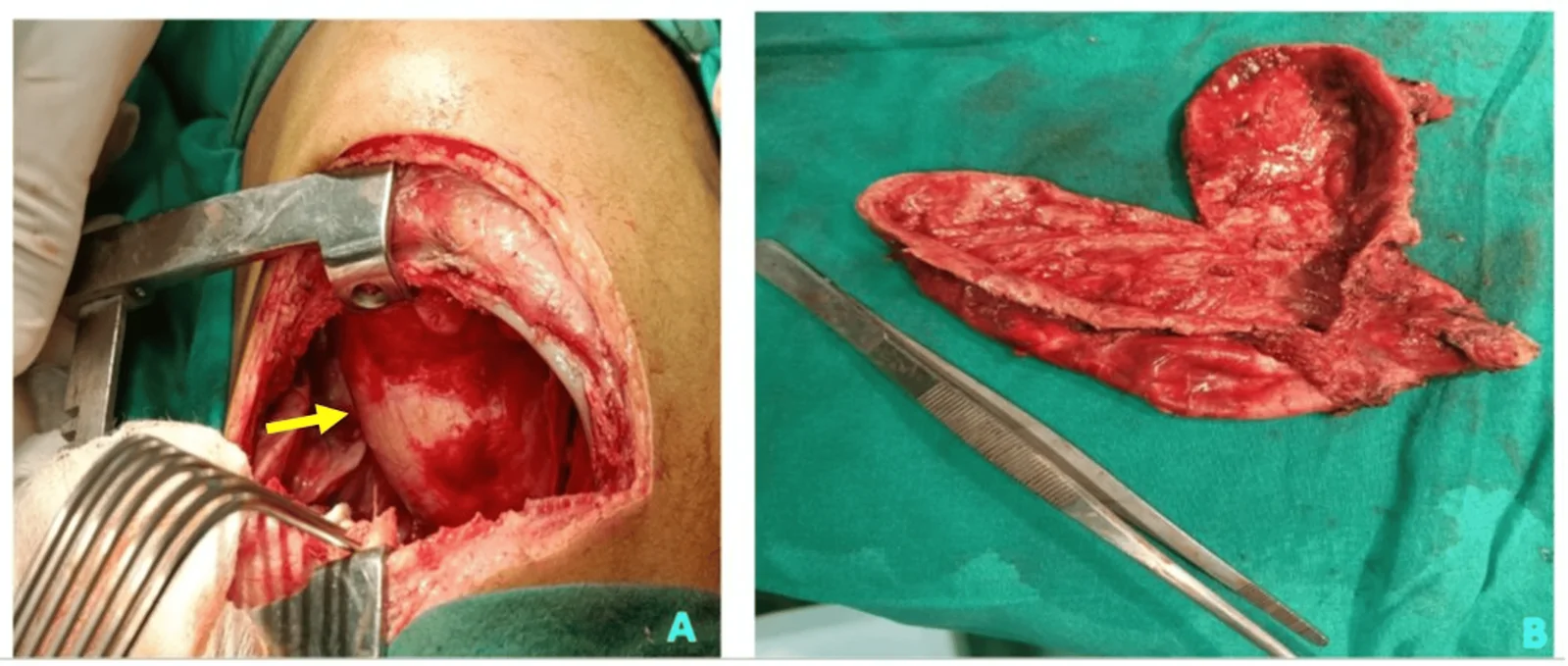
(A) Intraoperative view showing the posterior mediastinal cyst after thoracotomy, prior to complete excision (arrow). (B) Completely excised cyst opened longitudinally, demonstrating the cyst cavity.

A multidisciplinary meeting involving Pediatric Hematology-Oncology, Pediatric Surgery, and Radiology concluded that the mass required surgical excision for a definitive histopathological diagnosis. Before surgical excision, hemoglobin declined to 6.5 g/dL due to ongoing hemolysis, requiring two packed red blood cell transfusions for preoperative optimization, with no spontaneous improvement prior to ATT initiation. The patient underwent a right posterolateral thoracotomy through the fifth intercostal space. Intraoperatively, the cyst was located in the posterior mediastinum between the pericardium and the aorta and was densely adherent to the right hemidiaphragm. Approximately 250 mL of pus was drained from the cyst. The cyst was completely excised and sent for histopathology (Figure 2). A chest tube was placed, and the patient had an uneventful immediate postoperative course. The chest tube was removed on the sixth postoperative day, and he was discharged on the ninth postoperative day. 

**Figure 3:**
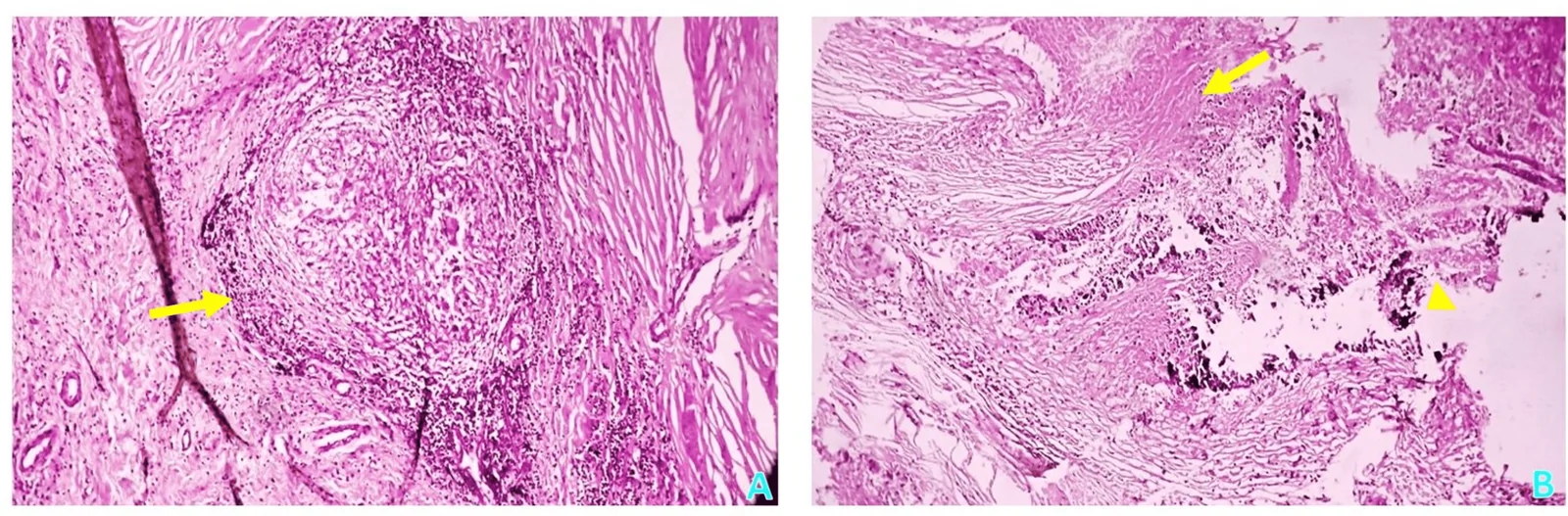
Histopathological sections showing: (A) a well-formed granuloma. (B) Necrosis (arrow) with dystrophic calcification (arrowhead) characteristic of tuberculosis (H&E stain, X100).

Gross histopathological examination revealed an unoriented, cut-opened cystic tissue measuring 13 x 7 x 2 cm with variable wall thickness. Microscopic examination showed well-formed granulomas with chronic granulomatous inflammation, calcifications, and necrosis (Figure 3).

The final histopathological opinion was a neurenteric cyst with chronic granulomatous inflammation consistent with TB. Stool GeneXpert for *Mycobacterium tuberculosis* was subsequently reported as negative; however, the histopathological evidence was deemed definitive.

Following complete cyst excision and initiation of antituberculous therapy, hemoglobin remained stable without further transfusion requirements. Corticosteroids were not given, as the AIHA was considered secondary to TB, with resolution anticipated upon treatment of the underlying disease. The patient was followed up monthly, with a six-month course of anti-tuberculous therapy (ATT) advised as per WHO recommendations, along with nutritional rehabilitation.

The patient was readmitted shortly thereafter for management of an infected wound, which resolved within a few days with appropriate wound care. On subsequent follow-up, the wound had healed, and the child was thriving, had a good appetite, and had significantly improved hematological parameters that remained stable without the need for any transfusion or immunosuppressive therapy.

## Discussion

This case describes a rare concurrence of three distinct clinical entities: a congenital neuroenteric cyst, secondary tuberculous infection of the cyst, and TB-associated AIHA.

Neuroenteric cysts arise from a developmental error during the third week of embryogenesis when the notochord fails to separate completely from the endoderm. They are frequently associated with vertebral anomalies, as seen in our patient with T1-T2 defects [3,4]. While these cysts typically present during infancy with respiratory distress or neurological symptoms due to mass effect, they may remain clinically silent until complicated by infection or progressive enlargement [3,5]. The chronic granulomatous inflammation, caseous necrosis, and dystrophic calcification identified in our patient's cyst confirm the unusual occurrence of secondary TB within a congenital neuroenteric cyst. Although secondary tuberculous infection of congenital mediastinal cysts is exceedingly uncommon, previously published reports have largely involved bronchogenic or other foregut cysts rather than neuroenteric cysts, underscoring the exceptional rarity of the present case [6,7]. We speculate that the enclosed cystic environment may have facilitated persistence of the organism by limiting effective immune clearance.

Radiologically, the large rim-calcified posterior mediastinal cyst closely mimicked a malignant mediastinal tumor, including lymphoma or a germ cell tumor. This underscores an important diagnostic challenge, as TB may mimic malignancy both clinically and radiologically. Normal serum alpha-fetoprotein and beta-human chorionic gonadotropin levels helped narrow the differential diagnosis; however, a definitive diagnosis was established only after surgical excision and histopathological examination. Previous reports have likewise emphasized that imaging findings alone are often insufficient to distinguish infected congenital mediastinal cysts from malignant lesions, making histopathological confirmation indispensable [6,7]. Furthermore, TB presenting as a mediastinal mass has been described even in immunocompetent children, reinforcing the need to consider TB in the differential diagnosis of mediastinal lesions in endemic settings [8].

We acknowledge, as a limitation, the absence of bacteriological or molecular confirmation of TB. Accordingly, this case is best classified as "clinically and histopathologically diagnosed tuberculosis," a category subsumed under the World Health Organization's "clinically diagnosed TB" classification [9], rather than bacteriologically confirmed TB, with the favorable response to ATT further supporting this diagnosis.

A particularly noteworthy feature of this case is the occurrence of Coombs-positive autoimmune hemolytic anemia. The proposed mechanism involves molecular mimicry, whereby *Mycobacterium tuberculosis *stimulates B-lymphocyte clones to produce antibodies that cross-react with erythrocyte surface antigens, resulting in immune-mediated hemolysis [10]. Our patient presented with severe pallor, jaundice, hepatosplenomegaly, a dimorphic peripheral blood smear, reticulocytosis, raised total and indirect bilirubin levels, increased LDH, and a positive direct Coombs test, fulfilling the diagnostic criteria for AIHA.

As reported in the literature, Coombs-positive hemolytic anemia, whether isolated or associated with other immune cytopenias, represents one of the rarest hematological manifestations of childhood TB [10,11]. Unlike primary AIHA, in which corticosteroids constitute first-line therapy, TB-associated AIHA requires eradication of the underlying infection to achieve sustained hematological recovery. Pediatric cases reported in the literature consistently demonstrate resolution of immune-mediated hemolysis following initiation of antituberculous therapy, with corticosteroids reserved for selected severe or refractory cases [10,11]. In our patient, hemolysis resolved following antituberculous therapy and complete surgical excision of the infected cyst, further supporting the concept that treatment of the underlying infection is the cornerstone of management in TB-associated immune cytopenias [10,11].

To the best of our knowledge, this is among the very few reported pediatric cases of TB involving a congenital neuroenteric cyst and appears to be the first reported pediatric case associated with Coombs-positive autoimmune hemolytic anemia. This case broadens the recognized clinical spectrum of extrapulmonary TB and congenital neuroenteric cysts, emphasizing that TB should remain an important differential diagnosis in children presenting with complex mediastinal cystic lesions, particularly in regions where TB is endemic.

## Conclusions

In TB-endemic regions such as Pakistan, maintaining a high index of suspicion for TB is paramount, even when the presentation is highly atypical. Owing to the variable sensitivity of the routinely performed GeneXpert test for TB, a proportion of cases may remain undetected on initial investigations, as in our case. This case demonstrates that TB can superinfect congenital anomalies such as neuroenteric cysts and present with rare systemic complications such as AIHA. A multidisciplinary approach and careful clinicopathological correlation are essential to avoid misdiagnosis, prevent unnecessary malignancy-directed therapies, and ensure the timely initiation of life-saving antituberculous therapy.
